# Chemical Characterization of an Encapsulated Red Wine Powder and Its Effects on Neuronal Cells

**DOI:** 10.3390/molecules23040842

**Published:** 2018-04-07

**Authors:** Diego Rocha-Parra, Jorge Chirife, Clara Zamora, Sonia de Pascual-Teresa

**Affiliations:** 1 Faculty of Engineering and Agricultural Sciences, Pontifical Catholic University of Argentina, Buenos Aires C1107AAZ, Argentina; diegofer2484@gmail.com (D.R.-P.); jchirife@gmail.com (J.C.); zamoramariacl@gmail.com (C.Z.); 2 National Scientific and Technical Research Council (CONICET), Buenos Aires C1425FQB, Argentina; 3 Department of Metabolism and Nutrition, Institute of Food Science, Technology and Nutrition (ICTAN), Spanish National Research Council (CSIC), E-28040 Madrid, Spain

**Keywords:** wine, polyphenol, encapsulation, HPLC-MS-QTOF, cell culture, neuronal, antioxidant

## Abstract

Red wine polyphenols are known for their implications for human health protection, although they suffer from high instability. For this reason, a red wine powder was prepared by freeze-drying encapsulation in maltodextrin/arabic gum matrix, and its composition was determined by means of high-performance liquid chromatography coupled quadrupole time-of-flight mass spectrometry (HPLC-MS-QTOF). More than thirty polyphenols, including anthocyanins, flavanols, flavonols, phenolic acids and stilbenoids, were identified. Some of the main quantified polyphenols were: malvidin-3-*O*-glucoside, malvidin 3-*O*-(6″-acetyl-glucose), petunidin-3-*O*-glucoside, quercetin-3-*O*-glucuronide, syringenin-3-*O*-glucoside, epicatechin, gallic acid and syringic acid. The biological activity of this de-alcoholized and encapsulated red wine on human neuroblastoma SH-SY5Y cells was studied. The results showed that the encapsulated red wine powder has active redox properties, as verified by performing reactive oxygen species (ROS) analysis utilizing a neuronal model. This could help explain its action against the neurotoxicity induced by 6-hydroxydopamine (6-OHDA).

## 1. Introduction

Polyphenols are secondary plant metabolites with diverse physiological functions in the plant, such as ultraviolet protection, attraction of pollinators, anti-parasite functions, and antioxidant protection [1]. Polyphenols are a complex group of compounds, and their analysis and identification has been addressed by several scientists by means of HPLC and UPLC-PDA-Q/TOF-MS in order to overcome this complexity [2,3]. In humans, polyphenols exert important functions through the activation of cellular antioxidant enzymes and signaling pathways [1,4].

Red wine polyphenols have historically been considered potent antioxidants, with a number of benefits for human health [5,6,7]. The available epidemiological studies suggest that moderate consumption of red wine is associated with a reduction in the risk of cardiovascular disease and carcinogenesis [8]. Regular and moderate red wine consumption is a frequently invoked factor for explaining the low incidence of cardiovascular events in France (known as “the French paradox”) compared with other industrialized countries [9,10,11,12].

Red wine polyphenols have vasorelaxant effects, which are associated with lower blood pressure [13]. Red wine, de-alcoholized red wine, and grape juice consumption have been shown to lower blood pressure in patients with coronary artery disease or hypertension [14,15]. These data suggest that polyphenols, rather than alcohol, are responsible for the vasorelaxant benefits.

With the current evidence on the biological value of red wine polyphenols and the deleterious effect of ethanol, a new interest has grown in the possibility of producing non-alcoholic wine extracts that nevertheless contain its polyphenols. It has been shown that the removal of ethanol from red wine has little impact on its beneficial health properties; namely, its antioxidant effects and protection against cardiovascular diseases [16,17]. One physical procedure to remove alcohol (and also water) is through wine dehydration, which leads to a concentrated wine dry extract containing its polyphenols. Freeze-drying has been proved to be the most suitable method for drying thermosensitive substances, minimizing thermal degradation reactions [18,19,20].

The biological activity of red wine polyphenols has been determined in some cell lines; however, only a few studies have been carried out on the effect of polyphenols on the nervous system or in neuronal cells [21]. In most of these studies, only isolated red wine polyphenols (mainly resveratrol) were studied. This approach ignores some studies suggesting that the health benefits attributed to wine polyphenols are due to their joint action rather than the action of individual compounds [22]. Furthermore, the complexity of the polyphenolic constituents of red wines makes it difficult to separate the specific contribution of each phenolic compound and cannot mimic the natural and balanced mixture of the hundreds of phenolic compounds present in red wine.

A de-alcoholized wine powder (WP) obtained via freeze drying (using suitable encapsulants), may serve to measure the biological effects of red wine polyphenols on cells, avoiding the presence of alcohol while maintaining the complex composition of red wine.

The purpose of this work was to determine, (a) the chemical composition of phenolics in the WP using high performance liquid chromatography coupled with high resolution Q-TOF mass spectrometry (HPLC-MS-QTOF), and (b) the biological activity of this dealcoholized wine powder on a human neuronal cell model.

## 2. Results

**Figure 1:**
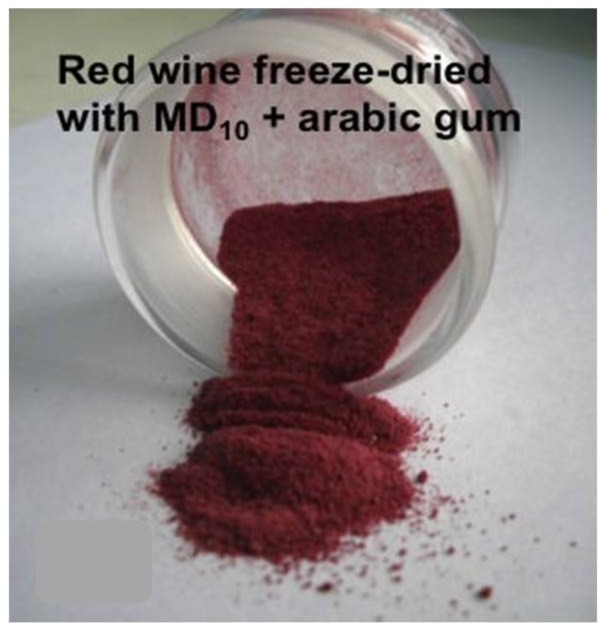
De-alcoholized freeze-dried red wine powder.

It has been previously determined that the WP used in this study is a free flowing de-alcoholized product (less than 1% residual alcohol) with good solubility in cold water that is easy to handle, shelf-stable (due to of its low water activity 0.11) and can be integrated as an ingredient in different food systems [18,19]. Figure 1 shows a picture of the WP. It has a total polyphenol concentration (Folin’s method) of 986 mg GAE/100 g powder (GAE, gallic acid equivalent) [23]. The increased polyphenol concentration (several times higher as compared to liquid red wine) resulted from the balance between water and alcohol elimination and MD_10_/gum Arabic gum incorporation.

The retention of total polyphenols after lyophilization/encapsulation of the liquid red wine was approximately 98% [18]. This agrees with the well-known fact that freeze-drying is a gentle method that is not expected to degrade polyphenols. During the freeze-drying of the present red wine, the maximum temperature in the sample was kept below 24 °C, which, in addition to the low partial pressure of oxygen (due to vacuum in the drying chamber), minimizes the oxidation of polyphenols. Some authors have determined the stability of several polyphenols in wine powder stored under accelerated storage conditions [19,20]. It is of note that due to its low water activity (a_w_ below 0.25), this WP was microbiologically stable at room temperature.

### 2.1. Red Wine Powder (WP) Polyphenolic Composition

Although some results on the composition of this encapsulated red wine have already been presented in the literature [19,20], a more complete characterization, as performed here, has not yet been made. Samples were prepared at a concentration of 50 mg/mL for their analysis by HPLC-QTOF. Analyses were done by using electrospray ionization (ESI) in the positive and negative mode. Thirty-five polyphenols, including anthocyanins, flavanols, flavonols, phenolic acids and stilbenoids were determined in the WP, as shown in Table 1. Malvidin-3-*O*-glucoside was the main compound in the anthocyanin group, while quercetin-3-*O*-glucuronide, epicatechin, gallic acid and piceid were the main compounds within Flavonols, Flavanols, Phenolic acids and Stilbenoids, respectively.

**Figure 2:**
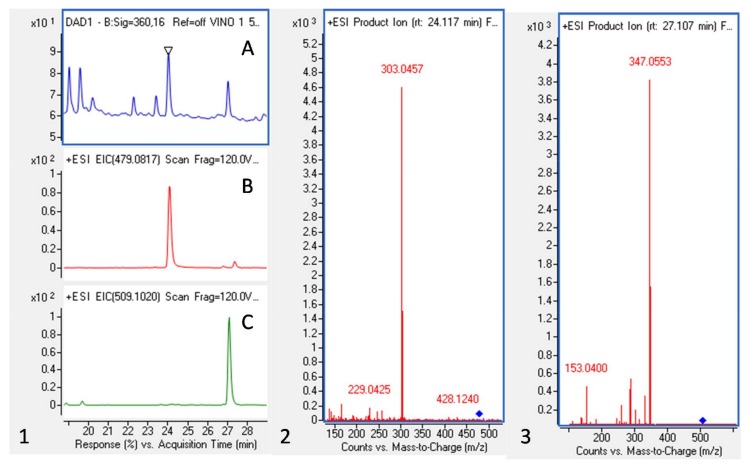
Ultraviolet–visible (UV-Vis) chromatogram (360 nm), extracted ion chromatograms (EIC) and MS^2^ spectra of two WP flavonols (quercetin-3-*O*-glucuronide and syrengenin-3-*O*-glucoside). 1-Chromatograms at 360nm (**A**), EIC at 479.0817 (**B**) and 509.1020 (**C**); 2-MS^2^ spectrum corresponding to the peak at MS+ 479.0817 with a retention time (Rt) of 24.1 and 2-MS^2^ spectrum corresponding to the peak at MS+ 509.1020 with a Rt of 27.0 (see Table 1 for identifications).

Figure 2 shows, as an example, the Ultraviolet–visible (UV-Vis) chromatogram at 360 nm and extracted ion chromatograms (EIC) of the mass spectrometry (QTOF-MS) analysis in the positive mode for ions 479.0817 and 509.1020 (left) and the tandem mass spectrometry (MS^2^) spectra of the two peaks corresponding to Quercetin-3-*O*-glucuonide and Syringenin-3-*O*-glucoside (right). Anthocyanins represented 80% of polyphenols quantified in the present analysis (Table 1). At 280 nm, one of the main peaks was identified in the negative mode as being gallic acid. Syringic acid and caffeic acid were also present in the WP, together with resveratrol and its glucoside, the piceid.

### 2.2. Cell Viability

In our study, it was found that pre-treatment of SH-SY5Y neuroblastoma cells with up to 200 μg/mL of WP and up to 200 μM of (−)-epicatechin did not affect cell viability. It was also verified that none of the encapsulating agents in WP (MD_10_ and Arabic gum) had a significant effect on cell viability.

To determine an appropriate dose of 6-hydroxydopamine (6-OHDA) treatment on SH-SY5Y cells, a dose-response curve was prepared. This resulted in a dose-dependent decrease in cell viability between 100 μM and 250 μM (from 100% to 37.8% respectively). Since 150 μM of 6-OHDA caused a decrease to about a half in cell viability (61.5%), this dose was used in the following studies to study the protective effect of WP and (−)epicatechin on 6-OHDA-induced cell damage.

**Figure 3:**
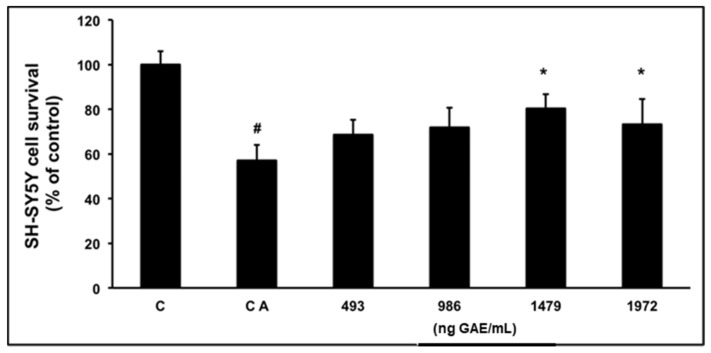
Protective effect of WP against 6-OHDA cytotoxicity in SH-SY5Y cells. SH-SY5Y cells were exposed to 6-OHDA (150 μM) in the presence of increasing concentrations of WP (from 50 to 200 μg/mL) corresponding to 494 to 1972 ng GAE/mL. Concentrations were expressed as ng gallic acid equivalent (GAE)/mL (9.86 ng GAE/μg WP). The control activated (CA) and control (C) bars mean that the cells were treated with and without 6-OHDA. The results are expressed as percentage of control and are represented by mean ± SD (*n* = 3). ^#^ *p* < 0.05 compared to the (C) group and * *p* < 0.05 compared to the (CA) group.

To determine the protective effects of WP and (−)-epicatechin on SH-SY5Y co-incubated with 6-OHDA, a cell viability assay was performed. The (3-(4,5-dimethylthiazol-2-yl)-2,5-diphenyltetrazolium bromide) tetrazolium (MTT) assay showed that the cell viability of untreated SH-SY5Y cells was decreased after 6-OHDA activation (57.1% of viability with respect to the control cells). However, the viability decrease was significantly reversed (*p* < 0.05) on SH-SY5Y when 6-OHDA was incubated with 150 and 200 μg/mL of WP, obtaining 88.3% and 73.3% of remaining surviving cells, respectively (Figure 3). On the other hand, cells co-incubated with 50 and 100 μg/mL of WP and 6-OHDA did not show any significant increase in SH-SY5Y viability (68.6% and 71.8% of cell viability respectively).

In the case of (−)-epicatechin, the results indicated that this compound (in a range of concentrations between 10 and 200 μM) significantly increased the cell survival rate to 81% and 102% (Table 2) compared to the activated control treated with 6-OHDA. These results indicate that WP and (−)-epicatechin are effective for the prevention of 6-OHDA-induced SH-SY5Y cell damage.

### 2.3. Effect of WP on ROS Production

**Figure 4:**
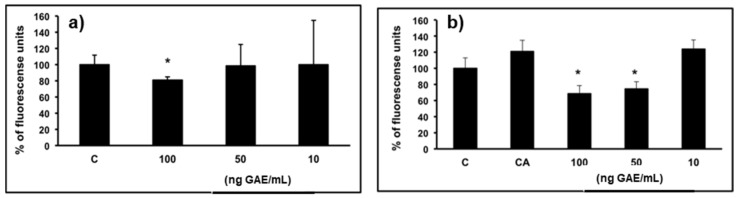
Protective effect of WP on intracellular ROS generation induced by *t*-BOOH treatment. SH-SY5Y cells were pretreated with increasing concentrations of WP (from 1 to 10 μg/mL) corresponding to 10 to 100 ng GAE/mL. Concentrations were expressed as ng gallic acid equivalent (GAE)/mL (9.86 ng GAE/μg WP). Values are expressed as a percentage relative to the control conditions. (**a**) Represents basal state (0 min of *t*-BOOH exposure) * *p* < 0.05 compared to the control group. (**b**) Represents *t*-BOOH activation (90 min of *t*-BOOH exposure). * *p* < 0.05 compared to the control activated group.

To establish whether the protective effects of WP were associated with inhibition of basal and *tert*-Butyl hydroperoxide (*t*-BOOH)-induced ROS formation in SH-SY5Y cells, a fluorescent probe-mediated assay was done. In the basal state, pre-treatment with 10 μg/mL of WP induced a significant (*p* < 0.05) reduction in cellular ROS (81%) generation compared to the control group (Figure 4). Regarding the effect of WP on the generation of ROS in a *tert*-butyl hydroperoxide (*t*-BOOH)-induced stimulus, pre-treatment with 5 and 10 μg/mL of powder significantly decreased ROS production (75% and 69%, respectively, with respect to the activated control). These results suggest that WP protects SH-SY5Y cells against induced oxidative damage.

## 3. Discussion

### 3.1. Red Wine Powder (WP) Polyphenolic Composition

Many authors have studied the polyphenolic composition of red wine from different grape varieties; however, very few studies exist on the actual composition of encapsulated red wine. In the present work, the presence of quercetin-3-glucuronide as one of the main flavonols should be noted (Table 1). Quercetin-3-glucuronide has already been detected in table grapes [24], although it is not so commonly found in European grape wine varieties or red wines. Additionally, the presence of syringetin-3-glucoside was found as one of the main flavonols in the WP, which is also somewhat peculiar for a Cabernet Sauvignon wine. This compound has only been reported, as far as we know, in Merlot and Sirah wines from Greece [25], and in different varieties of grapes used for “Vinho Verde” from Portugal [26]. Only a few authors have reported its presence in Cabernet Sauvignon grapes and wines from California [27].

Malvidin-3-glucoside represented 45% of total anthocyanins identified in the present analysis (Table 1). The encapsulated WP is also rich in flavanols; the monomers catechin and epicatechin and dimers B1 and B2 are especially abundant. Gallocatechin monomers and procyanidin trimers mainly formed by epicatechin and catechin units were observed. This is in accordance with the composition of most red wines [28].

In the WP, free anthocyanins in the form of 3-*O*-monoglucosides represent the predominant form within the Anthocyanins group (56%). In other studies, with *Vitis vinifera* wines, similar conclusions have been reached [29,30]. In addition, there are other works with Cabernet Sauvignon wines that found that the content of malvidin-3-*O*-(6-*O*-acetyl)-glucoside is considerable for this varietal, and in the case of WP, represents 81.7% of the Anthocyanins-3-*O*-(6-*O*-acetyl)-glucoside group [31].

### 3.2. Cell Viability

It is known that hundreds of polyphenols are present in red wine. Thus, there is not any specific reason to include epicatechin in the activity trial besides of the fact that (−)-epicatechin is the main compound in the flavanols group (see Table 1). However, this polyphenol has been mentioned in the literature for its positive biological effects in cells [32]. In the case of (−)-epicatechin, a greater cellular survival can be observed at lower concentrations compared to WP. This behavior can be explained because the final balance of the effect of the powder is due to the joint action of the complex composition of the WP. In other words, it is the result of the synergies and/or antagonism effect between the different compounds.

The SH-SY5Y cell model has been chosen by some authors to study the neuro-biological effect of some polyphenols. For example, some of them have shown that resveratrol induced activation of the mitogen-activated protein kinases, extracellular-regulated kinases (ERK1 and ERK2), in human neuroblastoma SH-SY5Y cells; others proved the anti-apoptotic effect of *trans*-resveratrol on paclitaxel-induced apoptosis; on the other hand, some researchers elucidated a neuroprotective effect of resveratrol against β-Amyloid (Aβ)-induced neurotoxicity and showed that resveratrol protects SH-SY5Y neuroblastoma cells from apoptosis induced by dopamine. In addition to this, studies have found that quercetin 3-glucoside protected SH-SY5Y cells against hydrogen peroxide-induced oxidative stress and can also attenuate monoamine oxidase-A (MAO-A) activity in that cellular model. Finally, some authors have tested different polyphenol-derived metabolites in dopaminergic models against oxidative stress-induced cytotoxicity [21,33,34,35,36,37,38,39,40].

Most of these studies on SH-SY5Y models only tested some of the compounds present in red wine (mainly resveratrol or *trans*-resveratrol). However, wine is a complex mixture, and it is likely that a multitude of chemical constituents, as well as their metabolites, might work synergistically to impact human health [41,42]. For this reason, a WP obtained from whole red wine was utilized and showed the ability to protect against 6-OHDA-induced cell damage on SH-SY5Y neuronal cells.

### 3.3. Effect of WP on Reactive Oxygen Species (ROS) Production

Reactive oxygen and nitrogen species are considered major determinants of many degenerative diseases. ROS can act by damaging biological structures, but they can also affect cell signaling by changing the redox cellular status or by participating in intracellular signaling [43]. Although the mechanisms accountable for the death of dopaminergic cells (SH-SY5Y) are not completely understood, multiple pieces of evidence from human post-mortem investigations and animal studies indicate that oxidative stress is an important role in initiating this process [44].

It has been reported that ROS and RNS (Reactive Oxygen and Nitrogen species, respectively) are considered to act as pro-inflammatory signals in vivo by stimulating the activation of tumor necrosis factor (TNF-a), interleukin (IL) IL-1b and IL6 genes through activation of the redox-sensitive nuclear factor (NF-kB) [45]. In this way, the reduction of ROS would contribute to activate a decrease in the signaling cascade when a cellular system has been affected by some toxic component.

The neuroprotection exerted by WP can be explained through its antioxidant capacity (via ROS), a result that points in the same direction as the in vitro observations made by Rocha et al. and Galmarini et al., who found antioxidant power in wine powder using different methodologies (i.e., the chromogen radical 2,2-diphenyl-1-picrylhydrazyl (DPPH*), β-Carotene/Linoleic acid assay and change in ferric reducing capacity, FRAP method) [19,20].

The results of the present work show that the WP has active redox properties in the neuronal model, which could help explain its action against the neurotoxicity induced by 6-OHDA. Overall, the results of this work can be explained by the combined effect resulting from the complex composition of red wine. Some studies have indicated that flavonols, in addition to their many other health benefits, contribute significantly to the protection of neuronal cells against oxidative-stress-induced neurotoxicity [46,47]. In Alzheimer’s disease, neuronal loss is preceded by the extracellular accumulation of amyloid-β peptide (Aβ). It has been shown that pre-treatment of primary hippocampal cultures with quercetin significantly attenuates Aβ-induced toxicity, lipid peroxidation, protein oxidation and apoptosis [46,47]. A dose-response study indicated that quercetin exhibited protective capacities against Aβ-induced toxicity by modulating oxidative stress at lower doses [46,47]. In cerebral ischemia, calcium dysregulation is one of the main instigators of neuronal cell death and brain damage [46,47]. Catechin may protect against the brain injuries produced by endogenous neurotoxins involved in the onset of Parkinson’s disease [46,47]. Catechin and epicatechin gallate have also shown an ability to suppress neuroinflammation and can attenuate and inhibit activation of microglia and/or astrocytes associated with the release of the mediators linked to the apoptotic death of neurons [46,47]. In addition, numerous studies indicate that catechin derivatives may delay the onset of neurodegenerative disorders such as Alzheimer’s disease through numerous different mechanisms, including iron chelators, radical scavengers, and modulators of pro-survival genes [46,47].

## 4. Materials and Methods

Standard polyphenols for HPLC-MS-QTOF analysis (phloroglucinol, gallic acid, catechin, (−)-epicatechin, quercetin, quercetin-3-*O*-glucoside and resveratrol were obtained from Sigma-Aldrich (Madrid, Spain). Delphinidin-3-glucoside, cyanidin-3-glucoside, peonidin-3-glucoside and malvidin-3-glucoside standards were obtained from Extrasynthese (Lyon, France), 6-OHDA, dichlorofluorescin (DCFH) and Dulbecco’s Phosphate Buffered Saline were purchased from Sigma-Aldrich Quımica S.A. (Madrid, Spain). HPLC-grade solvents were purchased from Merck (Darmstadt, Germany). Dulbecco Modified Eagle Medium‎ (DMEM), fetal bovine serum (FBS), penicillin/streptomycin, trypsin and l-glutamine were purchased from Lonza (Barcelona, Spain). Dimethyl sulfoxide (DMSO) was obtained from Panreac (Barcelona, Spain). Cell culture dishes were obtained from BD Falcon (Madrid, Spain).

The wine used was a commercial Cabernet Sauvignon, “Postales del Fin del Mundo” from Neuquén province, Argentina. Its alcohol content was 13.7% and its pH 3.6. Total polyphenol content was 2230 ± 160 mg GAE/L as determined by the Folin-Ciocalteau method [23]. The carbohydrates used for encapsulation were maltodextrin dextrose equivalent 10 (MD_10_) from Productos de Maíz, S.A., Argentina and gum arabic (from Gelfix, Buenos Aires, Argentina).

### 4.1. Encapsulation Procedure

In the present work, the encapsulation procedure described in previous works was followed [22]. A mixture of MD_10_ and gum arabic (65:35) was dissolved (9% total weight in red wine). The wine with dissolved encapsulating agents was poured into an aluminum tray (depth of sample, 1 cm), frozen and freeze dried. The freeze-drying process was performed using a laboratory-scale FIC148 LI-I-E300-CRT freeze dryer (Rificor, Buenos Aires, Argentina) operated with a freezing plate and condenser at −40 °C and at a vacuum of 100 μm Hg for 40 h at room temperature. Temperature of the dry layer did not exceed 25 °C.

### 4.2. HPLC-QTOF Analysis

Samples were prepared by weighting 50 mg of WP in methanol:water (1:3) containing formic acid at 0.1%. Prior to injection samples were filtered through a syringe filter (0.45 μm). Quantitative analysis of phenolic compounds was performed using an Agilent 1200 series liquid chromatographic system equipped with a quaternary pump, thermostatic autosampler, thermostated column compartment, and diode-array detector (DAD). High performance liquid chromatography coupled to quadrupole time-of-flight mass analyzer (HPLC-QTOF) with an electrospray ionization source (ESI) Jetstream technology was used. The spectra were recorded in negative and positive ion mode and the MS detector was programmed to perform a consecutive scan series: extended dynamic range, low 1700 *m*/*z*. A 5 μL aliquot of sample was injected and analyzed in a Phenomenex Luna C18 column (150 × 4.6 mm, 3 μm). Two solvents were used during the analysis. Solvent A was composed of distilled water/formic acid (FA) (0.1% FA) and solvent B consisted of acetonitrile with formic acid (ACN/FA) (0.1% FA). A constant flow of 0.5 mL/min was applied with the following gradient elution: (%B: 0 min 10%, 30 min 30%, 35 min 35%, 40 min 45%, 50 min 10%). The identification of compounds was done by comparing spectral characteristics of sample compounds, as well as by fragmentation, and comparison of the obtained fragments with the literature data. Standards of polyphenolic compounds were also injected to help in the identifications. An Agilent Mass Hunter Workstation was used for data acquisition and processing.

### 4.3. Cell Culture and Viability

SH-SY5Y human neuroblastoma cells were seeded and cultured regularly in DMEM containing 4.5 g/L glucose-584 mg/L l-glutamine and supplemented with 10% fetal bovine serum (FBS) and 1% penicillin/streptomycin (5000 U/mL). Cells were plated in 75 cm^2^ tissue culture flasks and maintained at 37 °C under 5% CO_2_ in a humidifier incubator until 80–90% confluence was reached. Medium was changed to serum-free medium 24 h prior to testing. Cells were harvested with trypsin–EDTA. SH-SY5Y cells were seeded in 96-well plates for the cytotoxicity test and cellular viability post 6-OHDA treatment (1 × 10^4^ cells per well), in 24-well plates for the ROS assay (1 × 10^5^ cells per well), and 6-well plates for the Nitric Oxide (NO) measurement (1 × 10^6^ cells per well).

SH-SY5Y cell viability was assessed by the (3-(4,5-dimethylthiazol-2-yl)-2,5-diphenyltetrazolium bromide) MTT reduction assay. Cell viability after WP and epicatechin pre-treatment was studied as follows. After 24 h of SH-SY5Y cell incubation (96-plate well), medium was replaced with serum-free medium containing different concentrations of WP (1 × 10^−3^–200 mg powder/mL) and (−)-epicatechin compound (10–200 µM, dissolved in dimethyl sulfoxide (DMSO), 0.1% final concentration) and then cells were incubated at 37 °C under 5% CO_2_ for 24 h. For all concentrations of WP, controls of encapsulating agents (MD10 and Arabic gum) were made. Control cells without treatment were incubated in serum-free medium without and with 0.1% DMSO for WP and (−)-epicatechin, respectively.

For cell viability after 6-OHDA treatment, cell medium was replaced (after 24 h) with serum-free medium containing different concentrations of 6-OHDA (25–250 µM dissolved in 0.02% ascorbic acid-PBS solution) and incubated at 37 °C under 5% CO_2_ for an additional 24 h (control cells were treated only with 0.02% ascorbic acid-PBS solution).

For cell viability after co-treatment with WP or epicatechin with 6-OHDA, cell medium (after 24 h) was replaced with serum-free medium containing: (a) different concentrations of WP (1 × 10^−3^–200 mg powder/mL), together with 150 µM of 6-OHDA or (b) different concentrations (−)-epicatechin (10–200 µM, dissolved in DMSO, 0.1% final concentration), together with 150 µM of 6-OHDA of 6-OHDA. The SH-SY5Y cells were incubated at 37 °C under 5% CO_2_ for another additional 24 h. Control cell samples were incubated in serum-free medium without and with 0.1% DMSO for WP and (−)-epicatechin, respectively and 0.02% ascorbic acid-PBS solution.

Thereafter, 20 μL of a MTT solution in PBS (5 mg/mL) were added to each well for the quantification of the living metabolically active cells after 2 h incubation. Then, formazan crystals in the wells were solubilized in 200 μL DMSO. Absorbance was measured at a wavelength of 570 nm employing a microplate reader PowerWave™ XS (BioTek Instruments, Inc., Winooski, VT, USA). The viability was calculated assuming that the controls for each respective test were 100% viable.

### 4.4. Evaluation of Reactive Oxygen Species (ROS) Generation

Intracellular ROS levels were quantified following a previously described method [48] with some modifications. SH-SY5Y cells were seeded in 24-well plates until they reached 80% confluence. Then, the cells were treated with different concentrations of WP (1–10 μg WP/mL). 24 h later, cells were washed with PBS and incubated with the culture medium and Dichloro-dihydro-fluorescein diacetate (DCFH-DA) probe for 30 min. Finally, plates were measured at two times: (a) Basal state: 0 min of *t*-BOOH exposure, and (b) *t*-BOOH activated state: 90 min of *t*-BOOH exposure. Measurements were made in a fluorescent microplate reader at an excitation wavelength of 485 nm and an emission wavelength of 530 nm (Bio-Tek, Swindon, UK).

### 4.5. Statistical Analysis

All the experiments were carried out in triplicate, and results are expressed as mean ± standard deviation. Results were analyzed by one-way analysis of variance (ANOVA) test using InfoStat v.2013 (Universidad Nacional de Cordoba, Argentina). Means comparisons among storage time were carried out by Tukey’s test at *p* < 0.05.

## 5. Conclusions

The polyphenol profile of a freeze-dried encapsulated red wine powder was determined using HPLC-QTOF analysis. Thirty-five polyphenols were identified, including anthocyanins, flavonols, flavanols, phenolic acids and stilbenoids. These results showed a rich and complex polyphenolic composition in the de-alcoholized/encapsulated red wine. The present study suggest that red wine powder exerts a neuroprotective effect against 6-OHDA-induced damage that can be explained by the action of wine polyphenols through an antioxidant mechanism as verified by performing ROS analysis.

## Figures and Tables

**Table 1:** Polyphenols present in encapsulated WP as identified by HPLC-QTOF.

Polyphenol	Rt (min)	M+	M−	Fragment	Abs Max (nm)	(mg/100 g WP)
Anthocyanins						249.92
Delphinidin-3-*O*-glucoside	7.8	465.10		303.04	520	5.90
Cyanidin-3-*O*-glucoside	9.9	449.10		287.05	520	0.73
Petunidin-3-*O*-glucoside	10.5	479.11		317.06	520	15.60
Peonidin-3-*O*-glucoside	12.7	463.12		301.06	520	6.06
Malvidin-3-*O*-glucoside	13.0	493.10		331.08	520	112.45
Delphindin-3-*O*-(6″-ac-glu)	15.0	507.08		303.02	520	4.33
Cyanidin- 3-*O*-(6″-ac-glu)	17.6	491.08		287.03	520	0.64
Petunidin- 3-*O*-(6″-ac-glu)	18.1	521.09		317.04	520	7.74
Peonidin- 3-*O*-(6″-ac-glu)	20.4	505.10		301.04	520	3.65
Malvidin 3-*O*-(6″-ac-glu)	20.5	535.11		331.06	520	72.87
Delphinidin 3-*O*-(6″-cou-glu)	22.0	611.13		303.04	520	0.24
Cyanidin 3-*O*-(6″-cou-glu)	24.0	595.14		287.05	520	0.15
Petunidin 3-*O*-(6″-cou-glu)	24.2	625.15		317.06	520	1.24
Peonidin 3-*O*-(6″-cou-glu)	26.6	609.13		301.05	520	1.48
Malvidin 3-*O*-(6″-cou-glu)	26.6	639.14		331.06	520	16.84
Flavonols						8.34
Quercetin-3-*O*-glucoside	23.1	465.10		303.04	360	0.27
Quercetin-3-*O*-glucuronide	24.1	479.08		303.04	360	4.19
Syringenin-3-*O*-glucoside	27.0	509.10		347.05	360	3.70
Syringenin- 3-*O*-(6″-ac-glu)	32.3	551.10		347.05	360	0.18
Flavanols						24.73
Gallocatechin	7.4	307.07		139.03	280	0.95
Epigallocatechin	10.3	307.07		139.03	280	0.18
Catechin	12.6	291.07		139.03; 123.03	280	5.06
Epicatechin	15.9	291.06		139.03; 123.03	280	5.75
Procyanidin B1	9.8	579.14		127.03; 409.08	280	5.18
Procyanidin B3	11.5	579.14		127.03; 287.04	280	1.56
Procyanidin B4	13.8	579.14		127.03	280	0.70
Procyanidin B2	14.1	579.14		127.03; 409.08	280	3.76
Procyanidin B7	18.4	579.11		127.03; 287.04	280	0.43
Procyanidin trimer EEC	12.3	867.21		289.07; 579.13	280	0.66
Procyanidin trimer EEE	17.1	867.16		291.05; 579.10	280	0.50
Phenolic acids						29.10
Gallic acid	5.3		169.01	125.01	280	16.75
Caffeic acid	15.6		179.03	135.04	280	1.97
Syringic acid	21.3		197.04	124.02; 169.02	280	10.37
Stilbenoids						0.15
Resveratrol	39.3		227.11	138.95; 185.05	280	0.07
Piceid (Res 3-*O*-glu)	28.5		389.22	227.06	280	0.08
Total Polyphenols by HPLC						312.23

Abbreviations: ac (acetyl), glu (glucose), B1 (epicatechin-(4β→8)-catechin), B3 (catechin-(4β→8)-catechin), B4 (catechin-(4β→8)-epicatechin), B2 (epicatechin-(4β→8)-epicatechin), B7 (epicatechin-(4β→6)-catechin), EEC (epicatechin-(4β→8)epicatechin-(4β→8)-catechin), EEE (epicatechin-(4β→8)epicatechin-(4β→8)-epicatechin), Res (Resveratrol).

**Table 2:** Protective effect of (−)-epicatechin against 6-OHDA cytotoxicity in SH-SY5Y cells. SH-SY5Y cells were exposed to 6-OHDA (150 μM) in the presence of increasing concentrations of (−)-epicatechin (from 10 to 200 μM). The results are expressed as percentage of non-treated control and are represented by mean ± SD (*n* = 3). * *p* < 0.05 compared to the control activated. Activated control were treated with only 6-OHDA (150 μM).

(−)-epicatechin (μM)	SH-SY5Y Cell Survival * (% of Control)
Activated Control	58.0 ± 5.5
10	81.1 ± 6.5 *
25	96.5 ± 4.8 *
50	99.4 ± 9.5 *
100	89.2 ± 10.9 *
200	102.8 ± 6.3 *
